# High-Sensitivity C-Reactive Protein Is a Predictor of Coronary Microvascular Dysfunction in Patients with Ischemic Heart Disease

**DOI:** 10.3389/fcvm.2017.00081

**Published:** 2018-01-12

**Authors:** David C. Tong, Robert Whitbourn, Andrew MacIsaac, Andrew Wilson, Andrew Burns, Sonny Palmer, Jamie Layland

**Affiliations:** ^1^Department of Cardiology, St. Vincent’s Hospital, Melbourne, VIC, Australia; ^2^Department of Cardiology, Peninsula Health, Melbourne, VIC, Australia; ^3^Department of Medicine, Monash University, Melbourne, VIC, Australia

**Keywords:** inflammation, C-reactive protein, microvascular dysfunction, myocardial injury, ischemic heart disease, index of microvascular resistance

## Abstract

**Background:**

Inflammation and microvascular dysfunction (MVD) are independently associated with adverse cardiovascular outcomes in patients with ischemic heart disease. This study aimed to assess the relationship between inflammation, MVD, and myocardial injury.

**Methods:**

Coronary microvascular function was assessed in 74 patients undergoing percutaneous coronary intervention (PCI) using the index of microvascular resistance (IMR) by a pressure–temperature sensor-tipped wire. Serum high-sensitivity C-reactive protein (hsCRP) level was quantified by rate turbidimetry. Severe MVD was defined as IMR ≥ 30. Pearson correlation was computed to assess the relationships between hsCRP, troponin, and IMR of culprit vessel. Predictors of severe MVD were assessed by regression analysis.

**Results:**

Acute coronary syndromes (ACSs) represented 49% of the total cohort. Study cohort was divided into low C-reactive protein (CRP) (hsCRP < 3 mg/L) and high CRP (hsCRP ≥ 3 mg/L) groups. There was higher representation of smokers (78 vs. 52%), diabetics (39 vs. 18%), and ACS (61 vs. 33%), as well as higher body mass index (29.4 ± 4.6 vs. 27.2 ± 4.1) in the high CRP group. Pre-PCI and post-PCI IMR were significantly elevated in the high CRP group compared to the low CRP group (pre-PCI IMR: 29.0 ± 13.9 vs. 17.4 ± 11.1, *p* < 0.0001; post-PCI IMR: 23.0 ± 16.8 vs. 15.5 ± 8.4, *p* = 0.02). Peak troponin levels were significantly raised in the high CRP group (9.96 ± 17.19 vs. 1.17 ± 3.00 μg/L, *p* = 0.002). There was a strong positive correlation between hsCRP and pre-PCI IMR (*r* = 0.85, *p* < 0.0001). Pre- and post-PCI IMR levels were correlated with peak troponin level (*r* = 0.45, *p* < 0.0001; *r* = 0.33, *p* = 0.005, respectively). Predictors of severe MVD include male gender (OR 3.0), diabetes (OR 3.7), smoking history (OR 4.0), ACS presentation (OR 8.5), and hsCRP ≥ 3 mg/L (OR 5.6).

**Conclusion:**

hsCRP is a significant predictor of MVD while MVD is associated with myocardial injury, supporting the central role of inflammation and MVD in the pathophysiology and complications of coronary artery disease.

**Clinical Trial Registration:**

Australian New Zealand Clinical Trials Registry (ACTRN): 12617000648325. Universal Trial Number (UTN): U1111-1196-2246.

## Introduction

The paradigm is shifting in our understanding of atherosclerosis and acute coronary syndrome (ACS). The observation that inflammation plays a major role in atherogenesis and cardiovascular outcomes has a significant impact on current management of coronary artery disease (1). Multiple risk markers have been evaluated in the past to improve risk stratification and prediction of adverse cardiac events (2, 3). Among myriads of inflammatory markers, C-reactive protein (CRP), an acute-phase reactant produced predominantly in hepatocytes, emerges as a valuable biomarker in refining risk assessment (4–6). However, conflicting data regarding its clinical utility in addition to current risk prediction models have hindered its widespread use in routine clinical practice (7–9).

The Coronary Microcirculation refers to small pre-arterioles and arterioles (<500 μm in diameter) that are the major determinant of coronary vascular resistance and modulates coronary blood flow in response to neural, mechanical, and metabolic factors (10). Microvascular dysfunction (MVD) is commonly considered a consequence of ACS secondary to distal embolization of atherothrombotic debris (11), as well as functional impairment of microcirculatory flow due to release of vasoactive factors and inflammatory mediators from the disrupted atherosclerotic plaques (12, 13). Conversely, MVD is associated with atherosclerotic disease progression, and it predates the clinical manifestation of myocardial ischemia (11). The presence of MVD is a predictor of long-term adverse cardiovascular outcomes even in those without significant epicardial disease (14–16). On the other hand, the systemic nature of MVD is evidenced by a diminished microvascular density in non-ischemic myocardium and presence of severe MVD in myocardium distant from the site of infarction (17, 18).

Both inflammation and MVD lead to adverse cardiovascular outcomes. To date, there is a paucity of evidence to suggest the link between inflammation and MVD despite this strong potential association. Therefore, this study will evaluate the relationships between inflammation, MVD, and myocardial injury.

## Materials and Methods

### Study Population

The study population consisted of consecutive patients undergoing percutaneous coronary intervention (PCI) for ischemic heart disease (IHD). Informed consent was obtained from the participants before coronary angiography. Patients were excluded if they had active inflammatory/autoimmune disorders, receiving immunosuppressant therapy, previous history of myocardial infarction, or PCI of the culprit vessel in the previous 12 months, previous coronary artery bypass graft surgery, severe renal impairment [estimated glomerular filtration rate (eGFR) < 30 mL/min], severe left ventricular dysfunction (ejection fraction ≤ 35%), contraindication to prolonged dual antiplatelet therapy, and significant valvular heart disease. ACS was defined as ST-segment elevation myocardial infarction (STEMI) or non-ST-segment elevation myocardial infarction (NSTEMI).

### Study Protocol

All patients had their blood samples taken upon initial presentation to hospital. Serum high-sensitivity C-reactive protein (hsCRP) levels were quantified by rate turbidimetry on a Beckman Coulter AU Analyzer. Study patients received an initial weight-adjusted bolus of 100 U/kg intravenous heparin with additional bolus dosing to maintain an activated clotting time of >250 s. Right femoral vein was cannulated and a 5F venous sheath was inserted to allow drug delivery.

A 6F coronary guiding catheter was used to engage the selected coronary artery. A standard dose of 200 μg of intravenous nitroglycerine was administered to each study artery to minimize changes in coronary volume. Coronary microvascular function was assessed using a dual sensor pressure–temperature wire. The guide wire was calibrated with distal sensor placed at the ostium of the coronary artery to equalize the guiding catheter pressure, and then advanced beyond the stenosis into the distal third of the vessel. Hyperemia was achieved by intravenous adenosine infusion at a rate of 140 μg/kg/min *via* right femoral vein. A physiological response to adenosine was observed in all patients.

Microvascular resistance of culprit vessel was assessed utilizing index of microvascular resistance (IMR) as previously described (19). Thermodilution curves and hyperemic transit time (Tmn_Hyp_) were derived by injecting 3 mL of room temperature saline into the coronary artery through the guiding catheter. IMR was calculated as the distal coronary pressure divided by the inverse of mean transit time at maximal hyperemia using the following formula (20):

$$\text{IMR}={\text{Pa}}_{\text{Hyp}}\times {\text{Tmn}}_{\text{Hyp}}({\text{Pd}}_{\text{Hyp}}-\text{Pw}/{\text{Pa}}_{\text{Hyp}}-\text{Pw})$$

where Pa was mean hyperemic aortic pressure and Pd was the mean distal coronary pressure. Pw was referred as the coronary wedge pressure, which was defined as the distal coronary pressure obtained during a 30-s balloon occlusion of the culprit vessel during the initial balloon inflation (21). Fractional flow reserve (FFR) was defined as the mean distal coronary pressure divided by the mean aortic pressure during hyperemia. Coronary flow reserve (CFR) was defined by dividing the baseline transit time by the hyperemic transit time (22). Care was taken to ensure that the distal sensor was in the same position between measurements to avoid errors in transit time acquisition. Significant MVD was defined as IMR > 30 in accordance with previously published data (23, 24).

Index of microvascular resistance, FFR, and CFR was measured in the culprit artery at baseline. Stenting of the culprit vessel was then performed and physiological measures were repeated. The decision to intervene and the use of glycoprotein IIb/IIIa inhibitors and direct thrombin inhibitors were at the discretion of operators. Troponin I and creatine kinase were sequentially measured every 6 h up to a maximum of 24 h following PCI.

The study protocol was approved by the Human Research Ethics Committee at St Vincent’s Hospital Melbourne. All subjects gave written informed consent in accordance with the Declaration of Helsinki.

### Statistical Analysis

Statistical analysis was performed using SPSS statistical software system. Continuous variables were summarized as mean ± SD and were compared with the Student’s *t*-test. Non-parametric tests were used where appropriate. Normality of data was assessed with the Kolmogorov–Smironov statistic. Logarithmic transformation of data was performed for non-normally distributed data. A Pearson product–moment correlation coefficient was computed to assess the relationship between hsCRP, peak troponin, and IMR of culprit vessels. Binary logistic regression analyses were used to investigate the predictors of severe MVD. Data are presented as odds ratios and 95% CI. *p* < 0.05 was considered statistically significant.

## Results

### Study Population

Consecutive patients admitted to hospital for PCI were screened for eligibility and a total of 74 patients were enrolled in the study. There were 36 ACS patients (9 STEMI and 27 NSTEMI) and 38 stable angina patients in the cohort. The study population included 66% smokers, 73% hypertension, 65% hyperlipidemia, 30% diabetes, 42% family history of IHD, and 23% previous acute myocardial infarction (AMI). Coronary angiography was performed *via* femoral access in 17 (23%) of patients.

Baseline, clinical, and angiographic characteristics of the cohort divided into low CRP (hsCRP < 3 mg/L) and high CRP (hsCRP ≥ 3 mg/L) were summarized in Table 1. There were no clinically significant differences in age, gender and traditional cardiovascular risk factors between the two groups, except history of smoking (52% in low CRP group vs. 78% in high CRP group, *p* = 0.02) and diabetes (18 vs. 39%, *p* = 0.048). Mean body mass index was also significantly different between the two groups (27.2 ± 4.1 vs. 29.4 ± 4.6, *p* = 0.03). All study patients except five STEMI patients were commenced on statin therapy before coronary angiography and physiology assessment. The high CRP group comprised more ACS patients compared with low CRP group (61 vs. 33%, *p* = 0.02). Target vessels and lesion characteristics were similar between both groups.

**Table 1:** Demographics and angiographic characteristics.

Variable	Low CRP (<3 mg/L), *N* = 33	High CRP (≥3 mg/L), *N* = 41	*p* Value
Age	59.3 ± 10.8	60.9 ± 10.9	0.53
Male	26 (79)	28 (68)	0.31
ACS presentation	11 (33)	25 (61)	**0.02**
BMI	27.2 ± 4.1	29.4 ± 4.6	**0.03**
Diabetes	6 (18)	16 (39)	**0.048**
Hypertension	23 (70)	31 (76)	0.57
Hyperlipidemia	22 (67)	26 (63)	0.77
Smoker	17 (52)	32 (78)	**0.02**
Family history of IHD	12 (36)	19 (46)	0.39
Prior AMI	8 (24)	9 (22)	0.82
**Target vessel**			
LAD	12 (36)	17 (41)	0.66
LCx	10 (30)	15 (37)	0.57
RCA	11 (33)	9 (22)	0.27
**Lesion characteristic**			
ACC/AHA A	9 (27)	7 (17)	0.29
ACC/AHA B	21 (64)	30 (73)	0.38
ACC/AHA C	3 (9)	4 (10)	0.92

*Numbers in the table refer to the absolute numbers of patients in each group. The numbers in the brackets refer to percentages. The bold values demonstrate statistically significant differences between variables in low CRP vs. high CRP groups*.

*AMI, acute myocardial infarction; BMI, body mass index; IHD, ischemic heart disease; LAD, left anterior descending; LCx, left circumflex; RCA, right coronary artery; ACS, acute coronary syndrome; CRP, C-reactive protein*.

Coronary physiological data are presented in Table 2. Mean IMR levels pre- and post-PCI were higher in high CRP cohort (29.0 ± 13.9 vs. 17.4 ± 11.1, *p* < 0.0001; 23.0 ± 16.8 vs. 15.5 ± 8.4, *p* = 0.02). All patients had TIMI 2 and TIMI 3 flow pre-PCI and underwent successful PCI with TIMI 3 flow at the conclusion of the procedure. No-reflow phenomenon did not occur post-PCI in any of our study patients. No major procedural and in-hospital complications were observed. There was no significant difference in relation to stent type, size, length, and number of stents deployed per patient.

**Table 2:** Coronary physiological measures and procedural details.

Variable	Low CRP (<3 mg/L), *N* = 33	High CRP (≥3 mg/L), *N* = 41	*p* Value
FFR pre-PCI	0.64 ± 0.18	0.64 ± 0.17	0.99
FFR post-PCI	0.94 ± 0.06	0.91 ± 0.06	0.05
CFR pre-PCI	2.03 ± 1.05	1.87 ± 0.98	0.51
CFR post-PCI	2.92 ± 1.81	2.41 ± 0.97	0.15
IMR pre-PCI	17.4 ± 11.1	29.0 ± 13.9	**<0.0001**
IMR post-PCI	15.5 ± 8.4	23.0 ± 16.8	**0.02**
Drug eluting stent, *n* (%)	44 (82)	16 (80)	0.89
Stent size, mm	3.18 ± 0.54	3.14 ± 0.51	0.74
Stent length, mm	25.4 ± 14.7	22.1 ± 9.1	0.27
Stent number/patient	1.3 ± 0.5	1.3 ± 0.6	0.77
Pre-dilated, *n* (%)	24 (73)	36 (88)	0.10
Post-dilated, *n* (%)	31 (94)	36 (88)	0.36
No. of inflations, *n*	4.9 ± 2.5	4.8 ± 2.5	0.86

*The bold values demonstrate statistically significant differences between variables in low CRP vs. high CRP groups*.

*FFR, fractional flow reserve; CFR, coronary flow reserve; IMR, index of microvascular resistance; PCI, percutaneous coronary intervention; CRP, C-reactive protein*.

Table 3 summarizes biochemical results between the two groups. Mean hsCRP level was higher in high CRP group compared to low CRP group (15.1 ± 19.3 vs. 1.4 ± 0.7 mg/L, *p* < 0.0001). Peak troponin levels were significantly elevated in high CRP group (9.96 ± 17.19 vs. 1.17 ± 3.00 μg/L, *p* < 0.0001). Serum levels of total cholesterol, low-density lipoprotein cholesterol, glycated hemoglobin (HbA1c), and eGFR were similar between the two groups.

**Table 3:** Biochemical results.

Variable	Low CRP (<3 mg/L), *N* = 33	High CRP (≥3 mg/L), *N* = 41	*p* Value
Peak troponin I, μg/L	1.17 ± 3.00	9.96 ± 17.19	**0.002**
Peak CK, U/L	159 ± 181	426 ± 866	0.06
Total cholesterol, mmol/L	4.9 ± 1.6	4.6 ± 1.2	0.42
LDL-C, mmol/L	3.1 ± 1.5	2.9 ± 1.0	0.46
HbA1c, %	6.1 ± 1.5	6.0 ± 0.9	0.70
eGFR, mL/min/1.73 m^2^	112 ± 34	96 ± 30	0.05
hsCRP, mg/L	1.4 ± 0.7	15.1 ± 19.3	**<0.0001**

*The bold values demonstrate statistically significant differences between variables in low CRP vs. high CRP groups*.

*LDL-C, low-density lipoprotein cholesterol; HbA1c, glycated hemoglobin; eGFR, estimated glomerular filtration rate; hsCRP, high-sensitivity C-reactive protein; CRP, C-reactive protein; CK, creatine kinase*.

### Correlations between IMR, hsCRP, and Cardiac Enzymes

**Figure 1:**
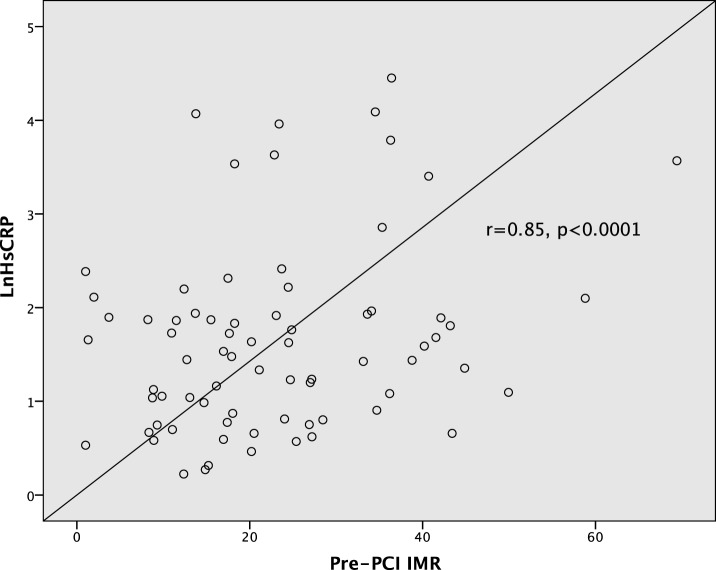
Correlations between high-sensitivity C-reactive protein and pre-percutaneous coronary intervention (PCI) index of microvascular resistance (IMR).

Baseline hsCRP level was positively correlated with pre-PCI culprit vessel IMR (Pearson *r* = 0.85, *p* < 0.0001) (Figure 1). This positive correlation was consistent in both ACS and non-ACS populations (*r* = 0.87 and *r* = 0.83, respectively, *p* < 0.0001). hsCRP level also correlated with post-PCI IMR (*r* = 0.38, *p* = 0.001) and peak troponin level (*r* = 0.44, *p* < 0.0001). Furthermore, there was positive correlation between peak troponin level with pre-PCI IMR (*r* = 0.45, *p* < 0.0001), and with post-PCI IMR (*r* = 0.33, *p* = 0.005). There were no statistically significant associations between hsCRP with age, gender, traditional cardiovascular risk factors, and angiographic characteristics.

### Predictors of Significant MVD

Using IMR ≥ 30 as cutoff threshold, patients with severe MVD (IMR ≥ 30) were compared against patients with normal microvasculature or mild MVD (IMR < 30). hsCRP level was significantly elevated in patients with significant MVD (16.0 ± 22.8 vs. 6.4 ± 11.6 mg/L, *p* = 0.02). Statistically significant difference in peak troponin levels was also observed between the two groups (12.6 ± 19.0 vs. 2.7 ± 8.2 μg/L, *p* = 0.003).

Table 4 demonstrates the predictors of severe MVD at baseline (using IMR > 30 as the threshold) by logistic regression analysis. ACS presentation was associated with a 8.5 times increased likelihood of impaired microvascular function while hsCRP level of ≥3 mg/L was associated with 5.6 times increased likelihood of severe pre-PCI MVD. In this study cohort, males were three times more likely to exhibit significant MVD. In addition, both smoking history and diabetes were also significant predictors of severe MVD (OR 4.0, *p* = 0.006; OR 3.7, *p* < 0.0001, respectively).

**Table 4:** Univariate predictors of severe microvascular dysfunction.

Covariates	Odds ratio	95% CI	*p* Value
Age	1.0	0.9–1.0	0.81
Male sex	3.0	1.1–8.3	**0.03**
Hypertension	1.0	0.4–2.4	1.00
Hypercholesterolemia	1.4	0.6–3.0	0.44
Diabetes	3.7	1.9–7.3	**<0.0001**
Smoking history	4.0	1.5–10.7	**0.006**
Acute coronary syndrome presentation	8.5	3.0–24.0	**<0.0001**
High-sensitivity C-reactive protein ≥3 mg/L	5.6	2.2–14.5	**<0.0001**

*The bold values demonstrate statistically significant predictors of severe MVD*.

**Figure 2:**
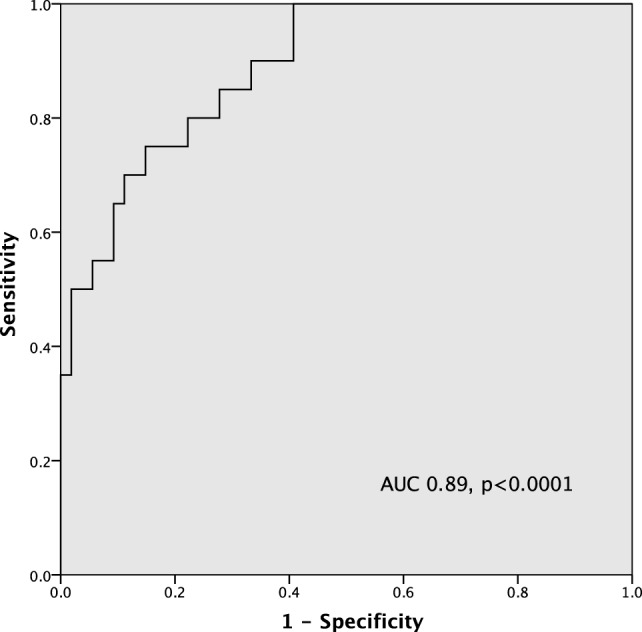
ROC curve of high-sensitivity C-reactive protein (hsCRP) in predicting severe microvascular dysfunction (MVD) (index of microvascular resistance > 30).

By ROC curve analysis, the optimal cutoff value for hsCRP in predicting significant MVD was 4.3 mg/L (AUC 0.89, 95% CI [0.81, 0.97], *p* < 0.0001) with a sensitivity of 85% and a specificity of 72% (Figure 2).

## Discussion

We demonstrate that hsCRP is strongly correlated with resting coronary microvascular status. Furthermore, both hsCRP and IMR are associated with degree of myocardial injury. Diabetes, smoking history, ACS presentation, and hsCRP ≥3 mg/L are important predictors of significant MVD.

### Inflammation and MVD

It is well known that inflammation plays a crucial role across all stages of atherosclerosis including plaque progression, rupture, and thrombotic complications (1), and CRP is deposited in the arterial intima at the sites of atherogenesis (25). Among patients with ACS, inflammation has been shown to be a systemic phenomenon that affects multiple vascular beds (26). In our study, we compared patients with high vs. low resting inflammatory state using hsCRP cutoff value of 3 mg/L as previous studies have consistently shown CRP’s association with adverse cardiovascular events above levels of 3 mg/L (27, 28).

In our study, patients with a heightened inflammatory state exhibited significant MVD at baseline and even after successful PCI. This could represent a “microvascular stunning” phenomenon similar to myocardial stunning, which could be a reversible and transient process driven by systemic inflammation. In fact, one previous study observed recovery of microvascular function on follow-up angiography after recanalization of a chronic total coronary occlusion (29). In addition, MVD could be a result of distal embolization of atherothrombotic debris or release of vasoactive and inflammatory mediators from the atherosclerotic plaques (11, 30, 31).

Several large-scale epidemiological studies show that elevation in markers of inflammation such as hsCRP prognosticates future cardiovascular events and poor outcomes, independent of traditional risk factors and across different populations (32, 33). On the other hand, it is unclear why patients with impaired microvascular function have adverse long-term cardiovascular outcomes (34), even in the absence of significant coronary artery stenosis (14–16). It may be that inflammation is the missing link between microvascular and macrovascular complications of cardiovascular disease.

### Inflammation, MVD, and Myocardial Injury

Inflammation predates the clinical manifestation of coronary artery disease and ACS. Numerous studies support the relationship between elevated levels of circulating inflammatory markers and adverse cardiovascular events (2, 35). Moreover, inflammatory response during and after a myocardial infarction plays a crucial role in repair and remodeling of damaged myocardium (36). We demonstrate an association between hsCRP level and peak troponin level (*r* = 0.44, *p* < 0.0001), which implies that there is a relationship between inflammation and periprocedural myocardial injury or tissue necrosis. We also show that peak troponin level is correlated with pre-PCI IMR. This observation is consistent with findings from a previous study which showed baseline microvascular function is an important determinant of periprocedural myocardial injury in patients undergoing elective PCI (37).

### Inflammation As a Common Link between Epicardial and Microvascular Disease

Studies have shown that MVD is an important predictor of adverse cardiovascular events (11, 14, 15). In congruence with this premise, our study indicates that inflammation could be the missing link between epicardial and microvascular disease. We have demonstrated positive correlations between baseline hsCRP, pre-PCI IMR, and peak troponin. In fact, inflammation may herald MVD, just as it contributes to plaque progression and thrombotic sequelae of ACS.

Both MVD and inflammation share a similar feature, of which their effect is systemic instead of being confined to a single vascular territory. Using positron-emission tomography, Uren et al. measured coronary vasodilator response by administering intravenous dipyridamole at 1 week after single-vessel AMI and reported the presence of MVD not only in infarcted myocardium but also in remote myocardium (18). This finding is further supported by a histopathological study which showed reduced microvasculature density in non-ischemic myocardium in patients who had a recent AMI (17). On the other hand, numerous studies confirmed the presence of widespread inflammation in ACS (38, 39), which extends beyond coronary vascular beds (26).

### Clinical Implications

It is important to appreciate the relationship between inflammation and MVD, as both factors are associated with heightened risks of future cardiovascular events and adverse outcomes. High baseline IMR is associated with increased cardiac death in STEMI patients (34) and is a predictor of periprocedural myocardial injury in elective PCI (37). Despite being a non-specific inflammatory marker, hsCRP could potentially be utilized as a surrogate marker to assess microvascular health or be incorporated into current risk prediction models to enhance discriminative value in identifying high-risk patients.

Prospective identification of patients with significant inflammatory milieu or microvascular dysfunction may allow clinicians to tailor the intensity of risk modification strategies. Patients with elevated hsCRP level may be offered intensive statin therapy as statins have been shown to exert pleiotropic effects on improving endothelial and microvascular function (40, 41). Inhibition of renin–angiotensin system should also be considered in high-risk population as it was shown to improve microvascular function (42).

The advent of knowledge in understanding the association between inflammation and MVD will likely give rise to development of novel agents and therapeutic strategies targeting inflammation and MVD. In fact, one such novel therapy include phosphodiesterase 5 inhibitor which has been shown to improve microvascular function in women who presented with ischemic symptoms but without obstructive coronary artery disease (43). Moreover, another recent study involving anti-inflammatory therapy with Canakinumab, a monoclonal antibody targeting interleukin-1β, demonstrated a reduction in recurrent cardiovascular events in patients with previous myocardial infarction and raised CRP but at a cost of more fatal infection (44). Many other anti-inflammatory therapeutic trials are currently underway including colchicine and methotrexate and will hopefully add to our armamentarium to treat cardiovascular disease.

### Study Limitations

While we have demonstrated the association between inflammation, MVD, and myocardial injury, this is still a relatively small sample. A strong correlation between CRP and IMR shown in our study is nevertheless not a proof of causation. In addition, CRP is a non-specific marker of inflammation and becomes elevated in a wide range of inflammatory conditions. There is also variability among individuals due to genetic polymorphisms, lifestyle factors, and medical comorbidities (45, 46). In this study, we excluded patients with history of active autoimmune or inflammatory disorders. Although widespread use of CRP to assess cardiovascular risk is not currently advocated, there is assemblage of clinical data to support CRP’s association with cardiovascular events and improving coronary risk prediction (28, 47).

Another study limitation is lack of comprehensive data on the duration and adherence of statin therapy before coronary physiology measurements. The intensity and duration of statin treatment may be confounding factors for the CRP vs. IMR relationship given the pleiotropic effects of statin in modulating inflammatory response and microvascular function (4, 41, 48). Nevertheless, there were no significant differences in the number of patients receiving statin therapy before IMR measurements in low CRP vs. high CRP groups or ACS vs. non-ACS groups.

## Conclusion

Inflammation is associated with MVD and myocardial injury and is a predictor of significant MVD. Inflammation may serve as a common link between epicardial and microvascular disease. Future studies are warranted to affirm this association and to establish the potential role of hsCRP measurement in tailoring risk modification strategies to manage cardiovascular disease.

## Ethics Statement

The study was carried out in accordance with the recommendations of St Vincent’s Hospital Melbourne Human Research Ethics Committee with written informed consent from all subjects. All subjects gave written informed consent in accordance with the Declaration of Helsinki. The protocol was approved by St Vincent’s Hospital Melbourne Human Research Ethics Committee.

## Author Contributions

DT and JL conceived the idea for the study, recruited patients, and wrote the initial draft of the manuscript. All other authors recruited patients and were involved in rewriting later versions of the manuscript.

## Conflict of Interest Statement

The authors declare that the research was conducted in the absence of any commercial or financial relationships that could be construed as a potential conflict of interest.
